# The Journey of Gender Identity in the Elderly: Needs and Challenges

**DOI:** 10.1192/j.eurpsy.2025.1726

**Published:** 2025-08-26

**Authors:** L. M. Carrasqueira, B. Wildenberg, P. Esteves, F. Tavares, B. Araújo, J. Brás, M. Coroa

**Affiliations:** 1 Psychiatry , ULS Região de Leiria, Leiria; 2 Psychiatry, ULS Coimbra, Coimbra; 3 ULS Algarve- Unidade de Faro, Faro, Portugal; 4 Psychiatry, ULS Algarve- Unidade de Faro, Faro; 5 ULS Coimbra, Coimbra, Portugal

## Abstract

**Introduction:**

Gender identity issues in the elderly are often overlooked in psychiatric practice. Older transgender and gender-diverse adults face significant barriers, including social isolation, stigma, and limited access to gender-affirming care. These challenges, coupled with healthcare discrimination and gatekeeping, negatively impact mental health outcomes, often leaving this population underserved in healthcare systems.

**Objectives:**

The aim is to identify the challenges transgender and gender-diverse elderly individuals face in accessing appropriate medical care and to explore how these barriers impact their overall health and well-being. Additionally, the objective is to propose strategies to improve both the mental health and general healthcare outcomes for this vulnerable group, ensuring that their specific needs are addressed within healthcare systems.

**Methods:**

A narrative review of the literature was conducted using PubMed, ResearchGate, and Medline databases. Search included combinations of the terms “gender identity,” “geriatic psychiaty “ and “gender dysphoria. Studies were selected based on their relevance to understanding the mental health and healthcare needs of elderly transgender and gender-diverse individuals.

**Results:**

The review revealed that elderly individuals with gender identity concerns experience higher levels of depression, anxiety, and social isolation. Historical discrimination and healthcare disparities significantly impact their well-being. Studies indicate that increased risks for dementia, linked to factors such as cardiovascular disease and sexually transmitted infections, further heighten their vulnerability.Moreover, there are significant gaps in gender-affirming care within geriatic services. Healthcare providers often lack the training necessary to address the specific needs of older transgender adults, leading to delays or denials in appropriate care.

**Conclusions:**

This review highlights that transgender and gender-diverse older adults remain a population often overlooked in psychiatric and geriatric care. Recognizing the importance of tailored care for this population is essential, as well as training healthcare providers and implementing gender-affirming treatments to ensure inclusive, equitable care that meets their specific needs.

**Disclosure of Interest:**

None Declared

